# Neurophysiological biomarkers of motor improvement from Constraint-Induced Movement Therapy and Robot-Assisted Therapy in participants with stroke

**DOI:** 10.3389/fnhum.2023.1188806

**Published:** 2023-09-15

**Authors:** Marcel Simis, Aurore Thibaut, Marta Imamura, Linamara Rizzo Battistella, Felipe Fregni

**Affiliations:** ^1^Instituto de Medicina Fisica e Reabilitacao, Hospital das Clinicas HCFMUSP, Faculdade de Medicina, Universidade de São Paulo, São Paulo, Brazil; ^2^Departamento de Medicina Legal, Bioética, Medicina do Trabalho e Medicina Física e Reabilitação, Faculdade de Medicina da Universidade de São Paulo, São Paulo, Brazil; ^3^Coma Science Group, GIGA-Consciousness, University of Liege, Liege, Belgium; ^4^Neuromodulation Center, Spaulding Rehabilitation Hospital, Harvard Medical School, Boston, MA, United States

**Keywords:** stroke, predictors, electroencephalography, power analysis, robot-assisted therapy, constraint-induced movement therapy

## Abstract

**Background:**

The mechanism of stroke recovery is related to the reorganization of cerebral activity that can be enhanced by rehabilitation therapy. Two well established treatments are Robot-Assisted Therapy (RT) and Constraint-Induced Movement Therapy (CIMT), however, it is unknown whether there is a difference in the neuroplastic changes induced by these therapies, and if the modifications are related to motor improvement. Therefore, this study aims to identify neurophysiological biomarkers related to motor improvement of participants with chronic stroke that received RT or CIMT, and to test whether there is a difference in neuronal changes induced by these two therapies.

**Methods:**

This study included participants with chronic stroke that took part in a pilot experiment to compare CIMT vs. RT. Neurophysiological evaluations were performed with electroencephalography (EEG) and transcranial magnetic stimulation (TMS), pre and post rehabilitation therapy. Motor function was measured by the Wolf Motor Function Test (WMFT) and Fugl-Meyer Assessment Upper Limb (FMA-UL).

**Results:**

Twenty-seven participants with chronic stroke completed the present study [mean age of 58.8 years (SD ± 13.6), mean time since stroke of 18.2 months (SD ± 9.6)]. We found that changes in motor threshold (MT) and motor evoked potential (MEP) in the lesioned hemisphere have a positive and negative correlation with WMFT improvement, respectively. The absolute change in alpha peak in the unlesioned hemisphere and the absolute change of the alpha ratio (unlesioned/lesioned hemisphere) is negatively correlated with WMFT improvement. The decrease of EEG power ratio (increase in the lesioned hemisphere and decrease in the unlesioned hemisphere) for high alpha bandwidths is correlated with better improvement in WMFT. The variable “type of treatment (RT or CIMT)” was not significant in the models.

**Conclusion:**

Our results suggest that distinct treatments (RT and CIMT) have similar neuroplastic mechanisms of recovery. Moreover, motor improvements in participants with chronic stroke are related to decreases of cortical excitability in the lesioned hemisphere measured with TMS. Furthermore, the balance of both EEG power and EEG alpha peak frequency in the lesioned hemisphere is related to motor improvement.

## 1. Introduction

Stroke is one of the main causes of mortality and disability worldwide, resulting in great economic and social burden (GBD 2016 Neurology Collaborators, 2019). Most of the therapeutic approaches for stroke rehabilitation rely on the concept that repetition of movements can induce a formation and consolidation of a new neuronal pathway via neuroplastic mechanisms. One example is Robot-Assisted Therapy (RT), which represents an effective treatment as it can deliver a high number of repetitive movements (Bertani et al., 2017). Another method for stroke rehabilitation is Constraint-Induced Movement Therapy (CIMT). This approach differs from most treatments as it restricts the unaffected arm to avoid use of the unaffected hand during intensive training with the affected upper limb (Thrane et al., 2014). Both CIMT and RT are effective methods to enhance motor function (Thrane et al., 2014; Bertani et al., 2017). However, they rely on likely different biological mechanisms, since CIMT may induce greater changes in the unlesioned hemisphere due to nonuse of the unaffected arm. Conversely, RT may induce more pronounced changes in the lesioned hemisphere due to the high number of repetitions performed with the paretic arm (Li et al., 2018). Although these are two of the most used therapeutic methods, their success remains limited.

To improve the available treatments, and to develop new approaches, it is essential to understand the mechanisms of stroke recovery using these techniques. Several models of brain reorganization after stroke have been proposed using previous data on functional magnetic resonance imaging (fMRI), positron emission tomography (PET), electroencephalography (EEG), magnetoencephalography (MEG), and transcranial magnetic stimulation (TMS) (Rossini et al., 1998; Pineiro et al., 2001; Calautti et al., 2003; Simis et al., 2016). One of the theories using this data is that the interhemispheric imbalance after a stroke may be the result of inadequate transcallosal inhibition (Murase et al., 2004). Recent studies have questioned the oversimplification of this model, proposing instead a bimodal balance–recovery model that combines the interhemispheric balancing with the concept of functional recovery of the structural reserve of the brain (Di Pino et al., 2014). Other authors go even further, suggesting that the interhemispheric imbalance is not due to poor motor recovery, but just a consequence of the underlying recovery processes (Xu et al., 2019). In addition, the understanding brain plasticity mechanisms contributes to the identification of biomarkers, and EEG and TMS are neurophysiological measurements with great potential for clinical use in this context (Aronson and Ferner, 2017).

Among the different types of biomarkers are diagnostic, predictive, prognostic, and the surrogate outcomes. The surrogate outcome is the biomarker that changes in correlation with clinical improvement, measuring the dynamic changes in brain reorganization. It has great importance since it allows to indirectly measure clinical progression, which can be useful to indirectly measure the effectiveness of the treatment and to identify the maximum potential for functional improvement (Simis et al., 2016; Aronson and Ferner, 2017; Thibaut et al., 2017; Simis et al., 2021).

Therefore, the understanding of the neuronal changes induced by two of the most frequently used therapies (i.e., RT and CMIT) may help to better explain these models. In this context, we analyzed the neurophysiological data (EEG and TMS) of a study that compared CIMT and RT in participants with chronic stroke. For this trial, 51 patients were enrolled with mild-to-moderate upper limb impairment. Based on our previous analysis, both groups improved on the Wolf Motor Function Test-Time (WMFT-T; mean change from 93.6 to 72 s), the Wolf Motor Function Test-Ability (WMFT-A; mean change from 3.6 to 3.93), and the Fugl-Meyer Assessment – Upper Limb (FMA-UL; mean change from 50.84 to 54.53 points), but there was no statistical difference between the groups for any of the variables (Terranova et al., 2021).

In the present exploratory study, we used TMS and resting-state EEG to study the neural mechanisms underlying functional improvement linked to CIMT and RT. We hypothesized that motor improvement is related to changes in EEG, mainly in the alpha and beta bands, and in TMS markers in the motor cortex. We also expected that these changes would be different in the CIMT group compared to the group who received RT. Our hypothesis is that RT may induce more pronounced changes in the lesioned hemisphere due to the high number of repetitions performed with the paretic arm, in comparison with CIMT. On the other hand, RT would induce less inhibition of activity in the unlesioned hemisphere in comparison to CIMT, due to the unuse of the health limb. Note that, during robotic therapy, the unaffected limb is also not trained; however, unlike CMTI, it is not restrained, which could justify a smaller effect on the unlesioned hemisphere.

## 2. Methods

This study was approved by the Ethics Committee for Analysis of Research Projects (CAPPesq) of the University of São Paulo Medical School, and written consent was obtained from all participants.

### 2.1. Sample characteristics

This study analyzed EEG and TMS data from the clinical trial registered on ClinicalTrials.gov (NCT02700061). The referred clinical trial tested the hypothesis that CIMT has better results than RT on upper limb motor recovery and functionality in participants with chronic stroke. The original trial included 51 participants who were randomized into two intervention groups: 36 sessions of RT (*N* = 25) or ten sessions of CIMT (*N* = 26), both associated with conventional therapy (Terranova et al., 2021). Of these 51 participants, 27 received neurophysiological (EEG and TMS) and clinical assessments pre and post-treatment. The data of these 27 participants are reported in the present study. The details of the intervention method, randomization and sample size calculation are better described in the main study from which this ancillary analysis was produced (Terranova et al., 2021).

Inclusion criteria: over 18 years of age, clinical and neuroimaging-based diagnosis of ischemic or hemorrhagic stroke, time since stroke from 6 to 36 months, clinically stable, and with minimal movement of the paretic upper limb (i.e., at least 20° of wrist active extension and at least 10° of metacarpophalangeal active extension). Exclusion criteria: muscle/joint damage or pain limiting the implementation of the therapy, progressive worsening of spasticity according to Modified Ashworth Scale, more than 1 stroke event, Mini-Mental Examination score lower than 20 points, psycho-affective disorders that prevented adherence to treatment, participation in another study protocol, and previous treatment with RT.

### 2.2. Clinical variables

FMA-UL was used to assess upper limb function, with scores ranging from 0 to 66 points (Fugl-Meyer et al., 1975). The WMFT was also used to measure proximal and distal upper-limb motor control on 17 functional tasks and can be scored by time to perform, ranging from 0 to 120 s (WMFT-T) and by the ability and quality of movement (WMFT-A), with an ordinal score from 0 to 5 for each of the 17 tasks (Pereira et al., 2011).

### 2.3. TMS variables

TMS data were acquired using 70 mm figure-8 coils (BiStim2, Magstim^®^ Company). The first dorsal interosseous muscle (FDI) was used to obtain motor evoked potentials (MEPs) from both hemispheres. The resting Motor threshold (MT) was defined as the lowest intensity of the stimulus that elicited a MEP with an amplitude of at least 50 μV in at least 50% of trials. The probable location of the FDI (called hotspot) was initially defined as the point on the coronal line with five centimeters between the Cz (10–20 EEG system) and the tragus of the ear. Subsequently the hotspot was confirmed by circular mapping around the starting point. Ten trials of MEP were collected for each hemisphere, using the intensity of 130% of the MT. The interval between each MEP measurement was at least 7 s. For data analysis, the average of the 10 MEP trials was calculated, using the MEP peak-to-peak amplitude.

### 2.4. EEG variables

EEG data were acquired using a 128-channel EEG cap with active electrodes (Acti-Champs, PyCorder, Brainvision LLC^®^). We only looked at the central electrodes (C3 and C4), which are related to the primary motor cortex for upper limb control. EEGs were recorded for 20 min during a resting state with the eyes closed. Then, the data were exported and analyzed offline with EEGLab (Delorme and Makeig, 2004) and MATLAB (MATLAB R2012a, The MathWorks Inc., Natick, MA, 2000). Each recording was filtered (0.5–40 Hz) and cleaned manually using EEGLab. We then averaged these values over different power bandwidths, including theta (4–8 Hz), alpha (8–13 Hz), low-alpha (8–10 Hz), high-alpha (10–13 Hz), low-beta (13–20 Hz), and high-beta (20–30 Hz). EEG Peak frequency was calculated for the alpha band. The EEG ratio was calculated by dividing EEG activity in the unlesioned versus the lesioned hemisphere.

### 2.5. Statistical analysis

The main statistical analysis was to identify the absolute change in neurophysiological biomarkers (EEG and TMS) that are related to motor improvement and to test the effect of the “type of treatment (RT vs. CIMT)” in the regression model. So, we initially performed univariate linear regression analyses for the three outcome variables (FMA-UL, WMFT-T and WMFT-A). Changes in motor function, EEG and TMS variables were calculated by subtracting the value obtained post-treatment minus pre-treatment. The independent variables were the “type of treatment (RT vs. CIMT)” and the neurophysiological data measured with EEG (e.g., high-beta in the lesioned hemisphere) and TMS (e.g., MT in the lesioned hemisphere). In a second step, we performed multivariate regression analyses using EEG and TMS variables in the same model. We determined the effects of the treatment group by adding the independent variable “type of treatment (RT vs. CIMT)” into the multivariate regression models. Variables were included in the model using the stepwise regression with forward selection approach. The EEG variables were included in the TMS model if the *p*-value was smaller than 0.10 and considered significant if *p*-value < 0.05. Due to the collinearity of the EEG data, more than one EEG variable was not included in the same model, and different models were built for each significant EEG variable. The assumptions of linear regression were tested (normality, linearity, homoscedasticity, and absence of multicollinearity) and the outliers that is influential point was excluded from the analysis. Besides, to determine the effects of confounders in these models, we added the clinical and demographic information as independent variables; the variables included were age, gender, time since stoke, stroke type (ischemic or hemorrhagic). It was considered confounder if the variable changed the β coefficient more than 10%.

Besides, to test the correlation between the improvement measured with FMA-UL and WMFT was used Spearman rank correlation test. It was used Wilcoxon Rank Sum Test to test the difference between the group (RT vs. CIMT), with the purpose of characterizing the sample. No correction for multiple comparisons was applied since this was an exploratory study and to minimize the risk of type II error. For the statistical analyses, we used Stata Statistical Software 15 (StataCorp LLC, Texas, USA).

## 3. Results

Twenty-seven patients were included [mean age 58.8 (SD: ±13.6), 13 females, 24 ischemic strokes, 8 right-hemisphere strokes, mean time since stroke of 18.2 months (SD ± 9.6)]. Participants had mild-to-moderate impairment, with a baseline score for FMA-UL of 53.2 (SD ± 7.1), for WMFT-T of 183.33 (SD ± 239.8), and for WMFT-A of 3.54 (SD ± 0.6). Thirteen individuals were allocated to the RT group, and 14 to the CIMT group. For TMS, 23 participants were analyzed (4 were excluded, 3 did not perform the post-treatment assessments, and 1 had technical problems during acquisition). The mean improvement was 0.42 points for WMFT-A, 2.59 points for FMA-UL, and 31.73 s for WMFT-T. The relation between FMA-UL and WMFT-T was negative (*p* = 0.010, Spearman’s rho = −0.4877). WMFT-T was also negatively associated with WMFT-A (*p* = 0.001, Spearman’s rho = −0.8262). No correlation between changes on the FMA-UL and the WMFT-A was found (*p* = 0.073, Spearman’s rho = 0.3503). Even with no correction for multiple comparisons, there was no statistically significant difference in improvement between groups (RT and CIMT) for the three clinical variables (FMA-UL, *p* = 0.450; WMFT-T, *p* = 1.000; WMFT-A, *p* = 0.645). The neurophysiological measurements at baseline, for the lesioned and unlesioned hemispheres, are summarized in Table 1; and the absolute change of the neurophysiological variables for the affected and unaffected hemispheres are summarized in Table 2.

**Table 1:** The EEG and TMS measurements at baseline for both hemispheres.

Variables (Baseline)	Lesioned (IQR)	Unlesioned (IQR)
TMS – MT (%)	51 (46–65)	49 (38–61)
TMS – MEP (μV)	1.06 (0.65–1.24)	1.19 (0.95–1.64)
EEG – Theta	0.40 (0.27–0.56)	0.46 (0.31–0.55)
EEG – Alpha	0.40 (0.33–0.60)	0.43 (0.34–0.61)
EEG – Low Alpha	0.54 (0.35–0.79)	0.58 (0.40–0.73)
EEG – High Alpha	0.35 (0.25–0.48)	0.37 (0.25–0.48)
EEG – Low Beta	0.20 (0.13–0.25)	0.21 (0.12–0.30)
EEG – High Beta	0.13 (0.05–0.15)	0.15 (0.06–0.18)
EEG – Alpha Peak	9.03 (7.89–9.93)	8.82 (7.84–9.80)

MEP, motor evoked potential; MT, motor threshold; IQR, interquartile range.

**Table 2:** The absolute change of the TMS and EEG variables for both hemispheres.

Variables (absolute change)	Lesioned (mean/SD)	Unlesioned (mean/SD)
TMS – MT (μ%)	1.61 (±4.7)	2.35 (±6.5)
TMS – MEP (μV)	0.07 (±1.1)	−0.12 (±1.0)
EEG – Theta	0.01 (±0.4)	−0.02 (±0.6)
EEG – Alpha	0.06 (±0.3)	0.03 (±0.4)
EEG – Low Alpha	0.19 (±0.6)	0.10 (±0.7)
EEG – High Alpha	0.01 (±0.2)	−0.03 (±0.2)
EEG – Low Beta	−0.07 (±0.1)	−0.10 (±0.1)
EEG – High Beta	−0.05 (±0.1)	−0.08 (±0.1)
EEG – Alpha Peak	−0.20 (±3.2)	0.20 (±3.9)

MEP, motor evoked potential; MT, motor threshold; SD, standard deviation.

### 3.1. Univariate analysis

We initially conducted univariate analyses to identify the variables that were associated with motor improvement indexed by FMA-UL, WMFT-T, and WMFT-A. We tested the effects of the treatment allocation, TMS variables (affected and unaffected hemispheres), and EEG power spectrum variables.

#### 3.1.1. Clinical vs. TMS variables

For the analyses with WMFT-T and FMA-UL as the dependent variables, none of the TMS variables were significant or reached the *p*-value < 0.10. For WMFT-A as the dependent variable, the absolute change of MT and MEP in the lesioned hemisphere were significant (*p* = 0.038, *β* = 0.043, *R*^2^ = 0.15; and *p* = 0.024, *β* = −0.23, *R*^2^ = 0.18; respectively), indicating that increases in MT and decreases in MEP are linked to motor improvement. These results are summarized in Table 3.

**Table 3:** Univariate analyses.

	Variables	WMFT-A
Lesioned TMS	MT (%)	***p* = 0.038**; *β* = 0.04; Adj *R*^2^ = 0.15
	MEP (μV)	***p* = 0.024**; *β* = −0.23; Adj *R*^2^ = 0.18
Unlesioned TMS	No variable	–
Lesioned EEG	No variable	–
Unlesioned EEG	High Beta	*p* = 0.092; *β* = −1.57; Adj *R*^2^ = 0.07
	Alpha Peak	***p* = 0.046**; *β* = −0.05; Adj *R*^2^ = 0.12
Ratio EEG	Theta	*p* = 0.085; *β* = −0.12; Adj *R*^2^ = 0.08
	Low Beta	*p* = 0.087; *β* = −0.20; Adj *R*^2^ = 0.08
	Alpha Peak	***p* = 0.036**; *β* = −1.18; Adj *R*^2^ = 0.13

WMFT-A as the dependent variable and the absolute change of the TMS and EEG variables for both hemispheres as independent variables. Variables with *p* < 0.1 was included in the table and variables with *p* < 0.05 are in bold. MEP, motor evoked potential; MT, motor threshold; *p*, *p*-value; β, beta coefficient; Adj *R*^2^, adjusted r squared; WMFT-A, Wolf Motor Function Test-Ability.

#### 3.1.2. Clinical vs. EEG variables

For the analyses with WMFT-T and FMA-UL as the dependent variables, none of the EEG variables were significant or reached the *p*-value < 0.10. For WMFT-A as the dependent variable, the absolute change of EEG alpha peak in the unlesioned hemisphere and the EEG alpha peak ratio were significant (*p* = 0.046, *β* = −0.053, *R*^2^ = 0.12; and *p* = 0.036, *β* = −1.18, *R*^2^ = 0.13; respectively), indicating that the decrease of EEG alpha peak in the unlesioned hemisphere is related to higher motor improvement. Moreover, the independent variables with *p*-value < 0.10 were the High Beta in the unlesioned hemisphere (*p* = 0.092, *β* = −1.57, *R*^2^ = 0.07), and the ratio of Theta and Low Beta (*p* = 0.085, *β* = −0.12, *R*^2^ = 0.08; and *p* = 0.087, *β* = −0.198, *R*^2^ = 0.08; respectively), indicating that the decrease of EEG Low Beta, High Beta, and Theta in the unlesioned hemisphere is related to higher motor improvements. These results are summarized in Table 3.

### 3.2. Multivariate analysis

#### 3.2.1. Treatment group (CIMT vs. RT)

To test the hypothesis that the motor improvements induced by the different therapies (CIMT and RT) would involve distinct neuroplastic changes, we added the variable “type of treatment (RT vs. CIMT)” to the univariate models described above (Clinical vs. EEG variables and Clinical vs. TMS variables). The variable “type of treatment (RT vs. CIMT)” was not significant and did not change the coefficients (β) of the neurophysiological variables.

#### 3.2.2. TMS and EEG models

Based on the results from the univariate analyses combining EEG and TMS in the same model, EEG variables were included in the model if their *p*-values in the univariate analyses were smaller than 0.10. For the models with the absolute change of MT in the lesioned hemisphere, the EEG bandwidth high alpha became significant in a way that the decreases in ratio (i.e., power increases in the lesioned hemisphere and decreases in the unlesioned) were related to superior clinical improvements, as measured with the WMFT-A (Table 4). For the models with the absolute change of MEP in the lesioned hemisphere, the absolute change of alpha peak in the unlesioned hemisphere, and the alpha peak ratio were significant (as univariable), in a way that increases in frequency in the lesioned hemisphere and decreases in the unlesioned were related to superior improvements in WMFT-A. For the analyses to determine the effects of confounders in these models, the variables gender, time since stoke, stroke type (ischemic or hemorrhagic) was not considered confounder or statistically significant. The variable age was considered confounder, since it changed the β coefficient more than 10% for the variable alpha peak ratio and the MEP in the lesioned hemisphere (Table 4).

**Table 4:** Multivariate analysis.

WMFT-A	β coefficient	*p*-value
**Model 1—Adj *R*^2^ = 0.27**		
High Alpha ratio	−0.432	0.049
MT (%) lesioned hemisphere	0.060	0.006
**Model 2—Adj *R*^2^ = 0.45**		
Alpha Peak ratio	−1.3	0.003
MEP (μV) lesioned hemisphere	−0.187	0.029
**Model 3—Adj *R*^2^ = 0.44**		
Alpha Peak Unlesioned	−0.061	0.004
MEP (μV) lesioned hemisphere	−0.234	0.007

Summarizes the three multivariate models. WMFT-A as the dependent variable and the absolute change of the TMS and EEG variables for both hemispheres as independent variables. Model 2 was adjusted for Age. MEP, motor evoked potential; MT, motor threshold; p, *p*-value; β, beta coefficient; Adj *R*^2^, adjusted r squared; WMFT-A, Wolf Motor Function Test-Ability.

## 4. Discussion

In this secondary analysis paper, participants with chronic stroke displayed upper limb functional improvements, as measured with the FMA-UL, WMFT-T, and WMFT-A, but with no differences between RT and CIMT. The present study focused on neurophysiological outcomes and had four main findings. First, changes in MT and MEP from the lesioned hemisphere have positive and negative correlations with WMFT-A improvements, respectively. Second, a decrease in the alpha peak from the unlesioned hemisphere and the absolute change of the alpha ratio are both negatively correlated with WMFT-A improvement. Third, in the model including MT changes, the decreases in ratio (power increases in the lesioned and decreases in the unlesioned) for high alpha bandwidth were correlated with superior improvements in WMFT-A. Fourth, the variable “type of treatment (RT vs. CIMT)” was not significant in the models. Moreover, motor improvements indexed by FMA-UL and WMFT-T were not predicted by any of the TMS and EEG metrics.

### 4.1. Univariate TMS analyses

The present longitudinal study, the decreases in cortical excitability in the lesioned hemisphere (measured by MEP and MT) are positively correlated with functional improvements, more specifically with improvements in WMFT-A. These results seem to contradict previous cross-sectional studies that showed negative correlations between MT from the lesioned hemisphere and motor function, as well as between motor improvements and increases in cortical excitability (Simis et al., 2016; Thibaut et al., 2017). However, this increase in MT is probably related to structural changes in corticomotor pathways combined with functional alterations in neural activity, rather than a maladaptive change (Rosso et al., 2017). We hypothesize that the present finding may be related to a reorganization of different inhibitory and excitatory pathways. Studies using MT and MEP showed a decrease in cortical excitability in the lesioned hemisphere, with a tendency to normalize from the acute to the chronic phase (McDonnell and Stinear, 2017). In the case of remaining severe impairment, an interhemispheric imbalance can remain (Simis et al., 2016).

On the other hand, previous studies using other neurophysiological measures, such as short-interval intracortical inhibition (SICI), which is an indirect measure of the GABA-A activity of interneurons, showed that stroke patients present a decrease of SICI in the affected hemisphere (compared to the unaffected hemisphere) in the acute phase, but not in the chronic phase, suggesting a reduction of inhibitory activity (GABA-A) in the acute phase, that tends to normalize in the chronic phase (Ziemann et al., 1996; McDonnell and Stinear, 2017). Similarly, studies using the cortical silent period (SP), which is related to GABA-B activity, showed an increase in SP duration in the lesioned hemisphere (compared to unaffected hemisphere) in the early phase post-stroke, and an inversion of this pattern in the chronic phase (Di Lazzaro and Ziemann, 2013; McDonnell and Stinear, 2017).

Therefore, MT and MEP are the results of a complex circuitry of inhibitory and excitatory activities that evolve between the acute and chronic phases following a stroke.

### 4.2. Univariate EEG analyses

In this study, we found that alpha peaks in the unlesioned hemisphere and the absolute change of alpha peak ratio (power in unlesioned/lesioned) have a negative correlation with WMFT-A improvement, suggesting that the increase of alpha peak in the lesioned hemisphere and its decrease in the unlesioned hemisphere are related to better motor improvement.

In the sensorimotor cortex, there is an overlapping EEG frequency for alpha and the *mu rhythms*, which is typically described as 8–12 Hz (Chatrian et al., 1959). Therefore, the decrease of alpha peak frequency could be interpreted as the decrease of *mu rhythm* peak frequency. The *mu rhythm* has been associated with somatosensory information, which increases in power during resting state and decreases with actual motor processing or mentalization of the movement (Yin et al., 2016). Previous studies have found a correlations between alpha activity in the motor cortex and motor improvement in stroke patients (Finnigan et al., 2007; Bentes et al., 2018). A hypothesis is that these changes may be related to the normalization of interhemispheric balance, suggestion the role of the normalization of alpha power for functional recovery.

### 4.3. Multivariate TMS and EGG analyses

By combining EEG and TMS in the same model, we found that increases in MT (i.e., decrease of cortical excitability) from the lesioned hemisphere, combined with decreases of EEG power bandwidth ratio (e.g;, ratio between the lesioned and unlesioned hemispheres) are related to better improvements as measured with the WMFT-A. We also found that decreases in MEP (i.e., a decrease of cortical excitability) from the lesioned hemisphere, combined with reductions of the alpha peak ratio (i.e., increase in the lesioned and decrease in the unlesioned hemisphere) and decreases of alpha peaks in the unlesioned hemisphere, are related to superior improvement in WMFT-A. Regarding our model, including MEP, MT, and alpha peak, the results are similar to the univariate EEG analyses, with a higher adjusted *R*^2^.

For EEG, the variables related to the ratio of unlesioned/lesioned hemisphere which was not significant in the univariate analyses, became significant when combined with the variable MT from the lesioned hemisphere. In this model, the decreases in the ratio (i.e., power increases in the lesioned and decreases in the unlesioned) of high alpha bandwidth is related to better clinical improvements as measured with the WMFT-A. These finding also support our hypothesis that motor improvement is related to a normalization of the interhemispheric balance of brain activity for several EEG bandwidths, as previously observed (Thibaut et al., 2017).

It is important to note that the mean absolute change of alpha power is positive for both hemispheres, meaning that alpha tends to increase, but in different magnitudes for the lesioned and unlesioned hemispheres. This is possibly related to a normalization of ERD/ERS mechanisms, as previously demonstrated (Pfurtscheller and Andrew, 1999). In the context of spinal cord injury, in which the motor deficit is bilateral, a recent study showed that the decreases in high-beta power and increases in ERD magnitude are associated with gait recovery (Simis et al., 2020). These findings suggested that the motor improvement is related to interhemispheric balance, but also with the balance of different neuronal activity within the cerebral hemisphere.

Unlike previous publications, beta activity was not statistically significant as a univariate nor in the multivariate model. This can be explained by the low statistical power of the sample. Furthermore, it is likely that the relationship between the beta band and the motor deficit depends on other factors (i.e., severity of the stroke, chronic pain, phase of the stroke), since the previous findings are not consistent. For example, in a previous retrospective cross-sectional neurophysiological study on stroke, were found that beta rhythm in the central region of unaffected hemispheres was positively correlated with motor function, while it negatively correlated with the beta rhythm in the affected hemispheres, as measured by FMA-UL (Thibaut et al., 2017). Moreover, another publication found that beta coherence in the unlesioned hemisphere had a negative correlation with FMA-UL (Simis et al., 2016). We highlight that, from the variables tested, only age was a confounder in the model, which may be explained by structural and functionally changes related to aging, what is reflected in neurophysiological measures (Inamoto et al., 2023). Also, the lack of significance of the variables tested as confounders may be related to the limitation of statistical power.

### 4.4. Clinical variables

The results showed a mean improvement in the 3 scales (0.42 points in WMFT-A, 31.73 s in WMFT-T, and 2.59 points in FMA-UL). Considering these values, the scales WMFT-A and WMFT-T showed an improvement above the “minimal clinically important differences” (MCID) (Lin et al., 2009). For FMA-UL, the improvement was below the MCID, which is defined as 5.25 points (Page et al., 2012). This small improvement measured by FMA-UL is expected for stroke patients in the chronic phase (Adeyemo et al., 2012). This is probably the reason why neither the TMS nor the EEG metrics could predict the changes measured by the FMA-UL.

For this study, we used FMA-UL and WMFT because these tests/scales are complementary, since FMA-UL measures mainly upper limb motor impairment and WMFT upper limb functional performance in specific tasks. We found that the improvements in WMFT-A were negatively correlated with the improvements in WMFT-T and FMA-UL, which can be related to the trade-off between speed and accuracy (Ammann et al., 2016). The decrease of time measured by the sub-item WMFT-T may be related to an improvement in movement precision measured by WMFT-A (Levin et al., 2009). This suggests that different aspects of motor improvement are related to distinct changes in brain function.

In the sample selected for this ancillary study, there was no statistically significant difference in clinical improvement between groups RT and CIMT, as the main study (Terranova et al., 2021). Moreover, the variable “type of treatment (RT vs. CIMT)” was not significant in the multivariate analyses. In our initial hypothesis, we expected a difference in brain activity induced by RT compared to CIMT, since they are based on different concepts of neural recovery. RT applies a large number of repetitive movements, which induce high activations of the lesioned hemisphere. On the other hand, CIMT decreases the recruitment of the unlesioned hemisphere, due to constraint of the unaffected upper limb. This finding suggests that different treatments have similar neuroplastic mechanisms of recovery. Moreover, patients in the conventional CIMT protocol also perform movements with the paretic hand and recruit the lesioned hemisphere, which may explain the similarity of changes in both groups (Thrane et al., 2014). It is likely that a difference between these 2 groups could be demonstrated in patients in the acute phase, which is characterized by greater motor improvements.

### 4.5. Motor networks connectivity and stroke recovery

Different techniques have been used to study motor network connectivity to determine measures such as intrahemispheric connectivity, interhemispheric connectivity, and network efficiency (Lee et al., 2019). One of the main models for stroke recovery is the theory of disrupted interhemispheric balance after stroke which is based mostly on trials using NIBS (Murase et al., 2004), in which techniques such as transcranial direct current stimulation (tDCS) and repetitive TMS are used to increase or decrease cortical excitability in the lesioned hemisphere or unlesioned hemisphere, respectively. Previous trials with NIBS showed that motor improvement was correlated with a decrease in cortical excitability in the unaffected hemisphere and an increase in the affected hemisphere, as well as with a reduction in transcallosal inhibition from the unaffected to the affected hemisphere, as measured with TMS (Fregni et al., 2006; Bolognini et al., 2011). Different from these studies, our results from TMS measurements did not show the same direction of changes. An explanation for this disparity is that studies with NIBS are inducing modification in different neuroplastic mechanisms in comparison to conventional treatments; and/or the modifications of cortical excitability with NIBS are related to the effects of the stimulation rather than to motor improvement. Even though studies with NIBS are normally performed in association with rehabilitation therapies, it is possible that increased cortical excitability in the injured hemisphere related to the effects of NIBS than with rehabilitation therapy (Fregni et al., 2006; Adeyemo et al., 2012; Fregni et al., 2021).

Moreover, the results from clinical trials with NIBS are very heterogeneous. A hypothesis for that is that the cerebral changes after stroke are more complex than the interhemispheric imbalance model and the neuronal modifications vary among patients (Di Pino et al., 2014; Xu et al., 2019). In this context, studies measuring transcallosal inhibition post-stroke with TMS suggested that suppressing the activity of the contralesional hemisphere could be beneficial for patients with good residual motor function, but not for patients with poor motor function (Bertolucci et al., 2018).

It is important to note that in our study, MT and MEP are linearly correlated with motor function. However, the average changes in MT and MEP were 1.33 (± 4.8) and − 0.05 (± 1.1), respectively, in a way that some patients who functionally improved presented neurophysiological changes in different directions, suggesting different neuroplastic behavior between patients. Thus, this difference in direction is probably related to the subtle and distinct reorganization of inhibitory and excitatory circuits, which may be better explained in future studies measuring other TMS related metrics such as SICI and SP. Moreover, the small magnitude of neuronal changes is related to the minor effect size of the motor improvement that occurred in the chronic phase of the stroke. A different clinical and neuroplastic behavior in the acute phase is expected, as discussed above.

The EEG power asymmetry between lesioned and unlesioned hemisphere has been described and seems to be correlated with the lesion volume and the severity of clinical symptoms (Sheorajpanday et al., 2009); however, these finds are not consistent between studies (Bentes et al., 2018). Our results showed that the motor improvement was related to the absolute change of alpha peak ratio, suggesting that subtle modification in EEG frequency activity can be related to important behavioral changes (as described in Table 2). Moreover, we showed that EEG power modification in the direction of improving interhemispheric asymmetry was related to motor improvement, which was significant only in the multivariate analyses combining TMS and EEG.

Therefore, motor improvement post- stroke seems to be related to complex mechanisms that are better explained by the association of different neurophysiological measurements. The understanding of these peculiarities is essential for the development of more efficient and customized treatments.

### 4.6. Limitation and future directions

Not consistent with our initial hypothesis, we did not find a difference in neurophysiological changes between CMIT and RT. However, we cannot conclude that the neuroplastic changes induced by both therapies are similar, since this study may be underpowered to answer this specific question due to the small sample size. Moreover, only patients with mild-to-moderate impairment in the chronic phase were included, which limited the external validity of our results (e.g., severe impairment and acute phase).

These findings may be useful for the development of new treatments such as brain-computer interfaces and could be used as biomarkers for motor recovery (Fugl-Meyer et al., 1975; Pereira et al., 2011; Xu et al., 2019). In addition, our findings should foster the search for new NIBS approaches that are not solely based on the theory of interhemispheric imbalance. One possibility is the use of alternating current stimulation to boost the cortical normalization activity.

## 5. Conclusion

The present results indicate that motor improvement, as measured with the WMFT-A, is correlated with an increase in MT and a decrease in MEP in the lesioned hemisphere. Furthermore, the decrease of EEGs markers (e.g., ratio between the lesioned and unlesioned hemisphere in the alpha band) is related to clinical improvement as measured with the WMFT-A. No neurophysiological variables tested were correlated with FMA-UL and WMFT-T. We did not find any differences between CMIT and RT neuroplastic changes.

## Data availability statement

The raw data supporting the conclusions of this article will be made available by the authors, without undue reservation.

## Ethics statement

The studies involving humans were approved by Ethics Committee for Analysis of Research Projects (CAPPesq) of the University of São Paulo Medical School. The studies were conducted in accordance with the local legislation and institutional requirements. The participants provided their written informed consent to participate in this study.

## Author contributions

MS and MI performed the data collection. AT cleaned and processed the EEG data. MS and AT performed the EEG analysis and the statistical analyses, and completed the first draft of the article. AT, MS, and FF interpreted the data. All authors designed the study, participated in the interpretation of the results, the writing of the manuscript, and the approval of its final version.
